# A Snapshot of Digital Transformation in Hepatology and Rare Liver Disease Related Health Care: A UK Perspective

**DOI:** 10.1016/j.mcpdig.2023.08.003

**Published:** 2023-09-16

**Authors:** Aftab Ala, James Liu Yin, Christopher Cussen

**Affiliations:** Institute of Liver Studies, King’s College Hospital NHS Foundation Trust, London, UK; Department of Gastroenterology and Hepatology, Royal Surrey County Hospital NHS Foundation Trust, Guildford, UK; Department of Clinical and Experimental Medicine, University of Surrey, Guildford, UK; Institute of Liver Studies, King's College Hospital NHS Foundation Trust, Denmark Hill, London, UK; Department of Gastroenterology and Hepatology, Royal Surrey County Hospital NHS Foundation Trust, Guildford, UK; Department of Clinical and Experimental Medicine, University of Surrey, Guildford, UK

Ebbert et al^1^ present an excellent assessment of models for collaboration between traditional and digital care providers with astute observations regarding the primary care landscape, where the greatest potential for direct competition and market disruption exists, and specialty care is where we will focus. Here, traditional providers hold an advantage over their digital counterparts by providing full diagnostic and management services, whereas digital providers must refer to multiple elements. As the authors state, opportunities exist for collaboration, but first there need to be models in place for equitable reimbursement.

Regulations surrounding data transfer and health care provision may present further barriers. In the United States, barriers exist for data transfer and health care delivery between states, which may hinder patient access to digital providers, particularly in less populated rural states where financial incentives to operate may be lower.

Like the United States, the UK health care regulatory framework is a patchwork of data protection and health care legislation. In the United Kingdom, the National Health Service digital provides the framework for digital transformation (DT): developing digital products and data services, working with health care providers and technology companies, and committing >$150 million to developing and testing artificial intelligence (AI) in pathology, radiology, and other specialties. Individual providers may develop or implement the technology and services they require.

We view the future of digital providers in specialty care as providing innovative products and services to traditional providers to enhance care. Using the authors’ example of inflammatory bowel disease, approaching half of patients with Crohn disease in the United States use biologic therapy,^2^ often requiring intravenous administration, which may complicate care provision in a health care environment, and may complicate care provision by digital providers. Digital providers might, as suggested by Ebbert et al,^1^ provide dietary advice and application-based support, but we would view these as outsourced services delivered on behalf of the traditional provider. A digital provider might also develop AI to improve efficiency and disease detection in endoscopy or medical imaging. We use hepatology and rare liver diseases as demonstrative examples for our argument.

Digital transformation generates impact in hepatology both through broader application in health care, for example, efficiency gains from electronic health care records, referral portals, or robotic process automation, and by the application of digital technology specifically to liver disease (Figure 1). Rare liver disease lends itself to DT, and particularly to AI, in developing novel tools in diagnosis and management. Increasing disease burden and mortality^3^ with incompletely elucidated pathogenesis of important liver diseases indicate significant unmet needs, whereas the huge volumes of demographic characteristics, biochemical, radiological, and histological data routinely collected in these patients mean that the data volumes required for AI development are likely acquirable. Artificial intelligence in the form of machine learning (ML) and deep learning (DL) is opening new frontiers in disease diagnosis, risk stratification, and management. The current focus in hepatology is on common liver diseases such as metabolic-associated fatty liver disease, but there are examples of technology being developed or adapted for rarer liver diseases.

**Figure 1:**
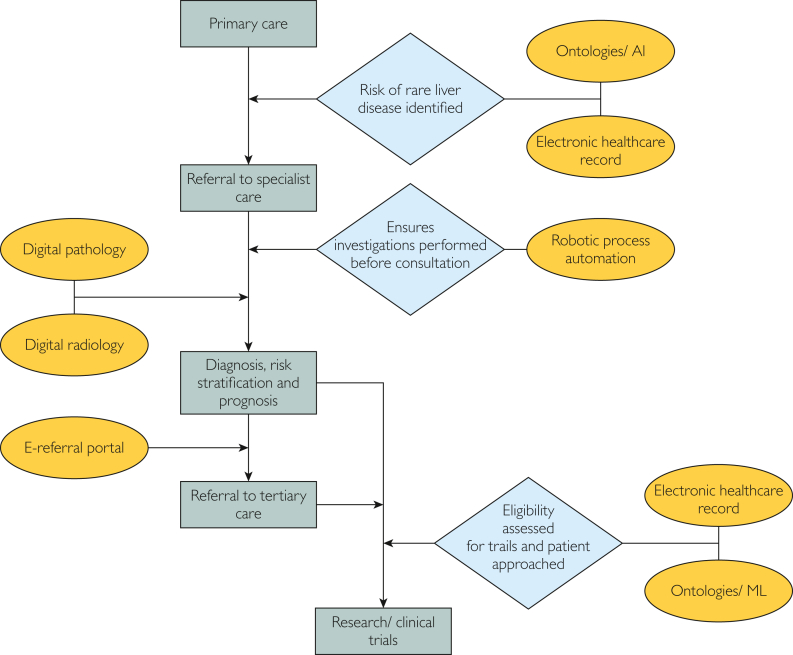
An Example of Digital Transformation in the Patient’s Journey.

### Radiology

Artificial intelligence in radiology has 2 categories: radiomics, using ML where a mathematical equation or series of equations are applied to images under close supervision, and DL systems, where convolutional neural networks extract information without the need for preprogramming. Digital companies are exploiting this technology to create innovative products such as Ferriscan (Resonance Health Limited), magnetic resonance cholangiopancreatography (MRCP+, Perspectum), and LiverMultiScan (Perspectum).

Ferriscan uses radiomics to quantify liver iron. Magnetic resonance images produced using a standardized protocol are transferred electronically to the digital provider, who applies a proprietary algorithm to accurately quantify liver iron content, with the result conveyed to the referrer. This has now replaced liver biopsy (with its associated risk and expense) in patients suspected of iron overload,^4^ thereby reducing costs for traditional providers both in diagnosis and the avoidance of unnecessary venesections, providing a clear incentive for collaboration.

In primary sclerosing cholangitis, radiological features may have prognostic value: early arterial peribiliary enhancement has been linked to higher risk scores and a poorer prognosis.^5^ Quantitative MRCP+ is an imaging tool able to create three dimensional quantitative models of the biliary and pancreatic systems, such as volumetric analysis, automatic detection of regions of variation, and number, length, and severity of strictures.^6^ The MRCP+ correlates well with validated risk scoring systems and could improve diagnosis and prognostic models in primary sclerosing cholangitis.

There has been some research into the use of multiparametric magnetic resonance imaging (LiverMultiScan) in autoimmune hepatitis (AIH) to produce iron-corrected T1 relaxation (cT1) maps, indicating areas of inflammation and fibrosis. There is evidence that a higher cT1 at diagnosis correlates with the risk of loss of biochemical remission and could therefore have prognostic value.^7^

In the future, DL may be able to extract data from medical imaging with as yet unrealized diagnostic or prognostic value across a range of liver diseases.

### Digital Pathology

Digital pathology is a rapidly growing area in the diagnosis of liver disease. Two forms exist: whole slide imaging and computational pathology (CPATH). Whole slide imaging uses a digital scanner to digitalize the entire histological specimen. When ML or DL is applied to whole slide imaging, it is termed CPATH. In diseases such as AIH where liver biopsy is essential in diagnosis, CPATH could be used to identify hereto unrecognized morphological features in AIH subtypes, which may aid diagnosis and differentiation between AIH and its mimics, such as drug-induced liver injury. Prognostic markers may be identified, allowing an application for CPATH in risk stratification and predicting future relapse. Future digital pathology companies might use proprietary algorithms to produce validated risk stratification or prognosis as seen in radiology. The use of AI for routine tasks and diagnoses will likely reduce costs and address workforce issues prevalent in pathology departments. Whole slide imaging allows easier and faster sharing of data between pathologists and referral to tertiary pathology providers. Its use remains uncommon though, particularly outside larger centers, partly owing to the availability and expense of the necessary digital scanning equipment.

### Big Data

Developments in big data and AI show promise in the detection and management of rare liver diseases. Allowing data linkage between electronic health care records in primary and secondary care could be used to create disease databases and identify new disease cases or eligible candidates for clinical trials. In primary biliary cholangitis, it has been shown that de novo cases and patients unknown to specialty care can be identified through the application of novel ontologies to primary care datasets.^8^ The large datasets needed to develop and validate AI technology are often held by traditional health care organizations. They reasonably view the data they hold as a commodity. This can be leveraged in collaborative arrangements with digital companies. An AI company called Deepmind accessed patient data at 1 organization in exchange for free use of its Streams application with technical support.^9^

## Conclusion

The models described by Ebbert et al^1^ on the basis of referral between digital and traditional providers to complement the strengths of each have merit and work for less complex care, but the barriers described by the authors are significant. We have discussed a more transactional landscape of service provision and collaboration: digital providers delivering services or licensing technology to traditional providers, and pecuniary reimbursement and anonymized patient data passing in the opposite direction. These relationships appear more mutually beneficial than multiple referrals between 2 providers delivering aspects of care for the same disease and provide more scope to work at scale within existing regulatory frameworks. If the hurdles can be overcome, the patient benefits realized through these collaborations could be transformative.

## Potential Competing Interests

The authors report no competing interests.
